# Healthcare insurance fraud detection using data mining

**DOI:** 10.1186/s12911-024-02512-4

**Published:** 2024-04-26

**Authors:** Zain Hamid, Fatima Khalique, Saba Mahmood, Ali Daud, Amal Bukhari, Bader Alshemaimri

**Affiliations:** 1 Department of Computer Science, Bahria University, Islamabad, Pakistan; 2 Faculty of Resilience, Rabdan Academy, Abu Dhabi, United Arab Emirates; 3 Department of Information Systems and Technology, College of Computer Science and Engineering, University of Jeddah, Jeddah, Saudi Arabia; 4 Software Engineering Department, College of Computing and Information Sciences, King Saud University, Riyadh, Saudi Arabia

**Keywords:** Unsupervised learning, Healthcare insurance, Healthcare insurance frauds, Association rules mining techniques

## Abstract

**Background:**

Healthcare programs and insurance initiatives play a crucial role in ensuring that people have access to medical care. There are many benefits of healthcare insurance programs but fraud in healthcare continues to be a significant challenge in the insurance industry. Healthcare insurance fraud detection faces challenges from evolving and sophisticated fraud schemes that adapt to detection methods. Analyzing extensive healthcare data is hindered by complexity, data quality issues, and the need for real-time detection, while privacy concerns and false positives pose additional hurdles. The lack of standardization in coding and limited resources further complicate efforts to address fraudulent activities effectively.

**Methodolgy:**

In this study, a fraud detection methodology is presented that utilizes association rule mining augmented with unsupervised learning techniques to detect healthcare insurance fraud. Dataset from the Centres for Medicare and Medicaid Services (CMS) 2008-2010 DE-SynPUF is used for analysis. The proposed methodology works in two stages. First, association rule mining is used to extract frequent rules from the transactions based on patient, service and service provider features. Second, the extracted rules are passed to unsupervised classifiers, such as IF, CBLOF, ECOD, and OCSVM, to identify fraudulent activity.

**Results:**

Descriptive analysis shows patterns and trends in the data revealing interesting relationship among diagnosis codes, procedure codes and the physicians. The baseline anomaly detection algorithms generated results in 902.24 seconds. Another experiment retrieved frequent rules using association rule mining with apriori algorithm combined with unsupervised techniques in 868.18 seconds. The silhouette scoring method calculated the efficacy of four different anomaly detection techniques showing CBLOF with highest score of 0.114 followed by isolation forest with the score of 0.103. The ECOD and OCSVM techniques have lower scores of 0.063 and 0.060, respectively.

**Conclusion:**

The proposed methodology enhances healthcare insurance fraud detection by using association rule mining for pattern discovery and unsupervised classifiers for effective anomaly detection.

## Introduction

The healthcare system plays a crucial role in maintaining the health and well-being of society, and many countries provide health insurance to their citizens to ensure they have access to medical care when needed. Health insurance can be provided by both public and private entities, and it helps to cover the cost of medical treatments, procedures, and medications. This system also helps to protect people from the financial burden of unexpected medical expenses that can arise due to illness or injury. The Sehat Sahulat Program was a health insurance initiative launched by the government of Pakistan in partnership with provincial governments, aimed at providing health coverage for needy people to minimize or eliminate out-of-pocket expenses and reduce poverty [1]. The program covers emergency and inpatient services requiring secondary and tertiary care but does not include outpatient services. The financial range for overall treatment coverage varies from 720,000 to 1,000,000 PKR and includes transportation for maternal care, referrals to tertiary care, and funeral allowances [2]. Similarly United States has its own Federal Government sponsored national healthcare program, Medicare, which provides affordable health insurance to individuals 65 years and older, and other select individuals with permanent disabilities [3]. Other than United States, countries like Canada, UK, France and many other also provide such facilities to their citizens. Advancements in medical sciences and technology have led to significant improvements in the health and well-being of the general public. However, the cost of quality healthcare can be high, and this is where health insurance plans play their role. Despite the significance of health insurance plans, fraudsters continually develop sophisticated schemes to evade detection. They may employ advanced techniques such as identity theft, billing for services not provided, or collusion among healthcare providers. Healthcare insurance frauds are causing billions of dollars loss in healthcare funds around the world. In 2010, the cost went up to 10% of total health care expenditure worldwide [4]. According to some reports, the US healthcare system loses around $505 billion to $850 billion every year. This percentage is from 9% to 19% of the total healthcare expenditure [5]. It can be easily seen that this additional burden leads to increased taxes and higher health insurance plans for individuals.

**Fig. 1:**
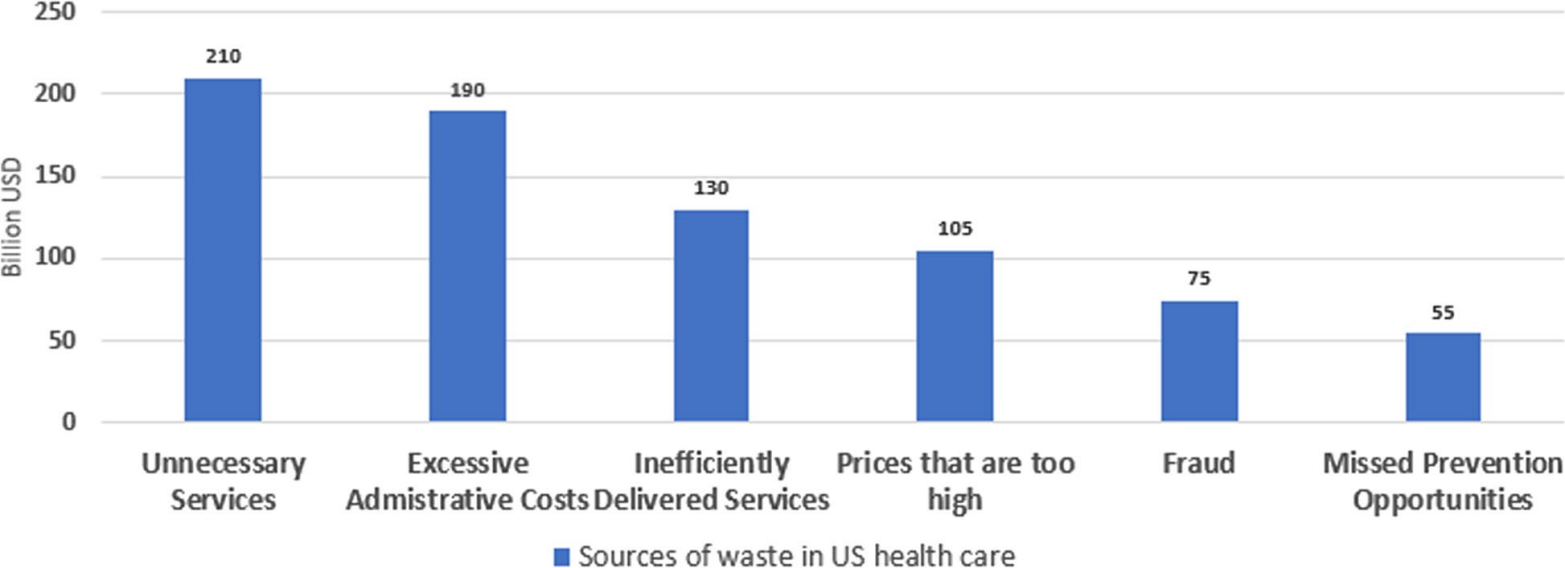
Sources of Waste in US Health Care

The Government Accountability Office of the United States (U.S. GAO) estimated over $51 million Medicare beneficiaries in 2013 with services costing somewhere over $600 billion. On the other side, it also costs $50 billion in improper payments, including some basic technical and human errors, which there may be fraudulent cases [6]. According to RGA data, published in 2017,The countries of Australia, Singapore, Malaysia, Thailand, the Philippines, Vietnam, and Korea, as well as Japan and Indonesia also faces healthcare fraud issues [7]. As per limited figures, the European continent has at least €56 billion in losses annually over fraud practices [8]. The Swedish insurance industry pays out SEK 70 billion loss to its customers in more than $3 million in claims; unfortunately, 5% of these payments turn out as fraudulent [9].

Insurance scams in the healthcare industry are resulting in losses of billions of dollars for public healthcare systems all over the world. Healthcare systems generate vast amounts of data, including patient records, billing information, and medical claims. Analyzing this large volume of data to identify anomalies or patterns indicative of fraud can be challenging and time-consuming. anomaly detection has been studied in different domain for identification of abnormal behaviors [10, 11] .Data mining techniques in combination with different analytical approaches i.e., machine learning techniques are now recognized as a key practice to identify fraud [12].

The Fig. 1 explains the most popular classification of the frauds in healthcare insurance system. Fraud can be identified through the services availing as well as providing patterns. Availing patterns such as repetition of services, age inconsistency, gender inconsistency, and visit frequency can leads towards fraud and waste of healthcare insurance. These patterns are performed by the patients. On the sides hospitals, providing patterns such as Billing, Unnecessary treatments, unnecessary procedures, charging multiple times, and misuse of credentials can leads towards fraud and abuse of system [13]. Recently researchers [14] tried to find behavioural relationship of different visits of patients utilizing hierarchical attention mechanism in fraud.

**Fig. 2:**
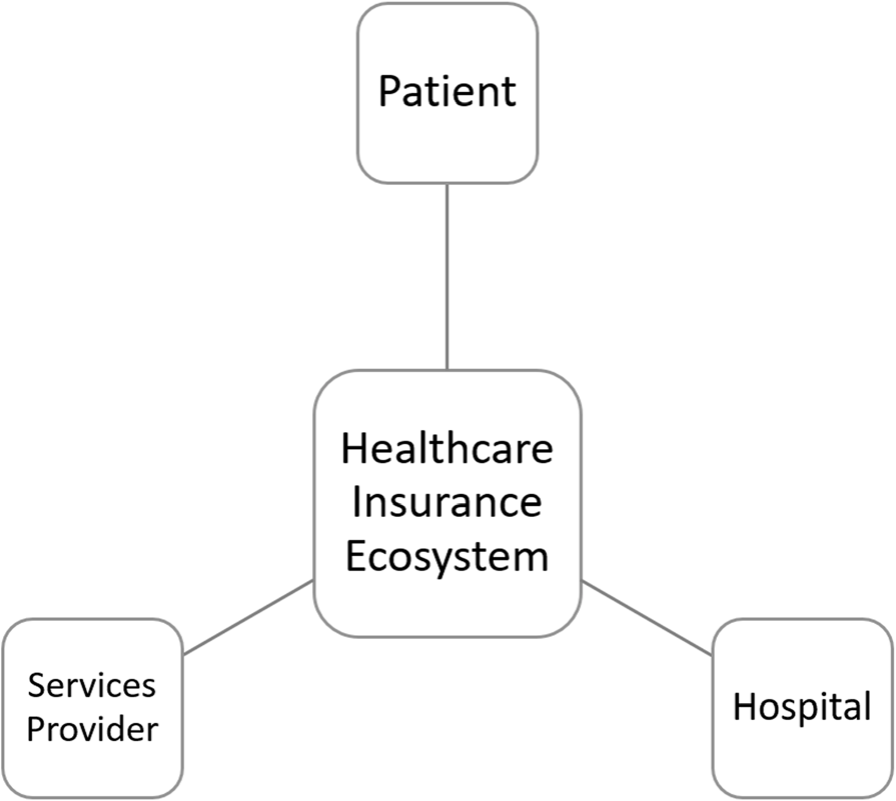
Healthcare Insurance Ecosystem :Patient, Hospital, and Services Providers

The healthcare insurance system involves mainly three actors - the insured, medical institutions, and insurance providers as depicted in the Fig. 2. Each actor may have different interests that can lead to fraud, for example, over-diagnosis and treatment by hospitals, fake medical treatment by insured individuals, and insufficient review of medical insurance settlement data by the health insurance providers. These frauds cause a significant loss to the insurance fund and threaten its normal operation. Measures should be taken to detect and report fraud, waste, and abuse in the system, including errors and abuse by providers, unnecessary costs to the payer, and exploitation of weaknesses in internal control mechanisms. Recently authors [15] proposed a Bayesian belief network based model to identify fraudulent activities involving all stakeholders in a transaction.

“Fraud, Waste, and Abuse” (often abbreviated as “FWA”) is a term used in the healthcare industry, including health insurance, to refer to practices that result in unnecessary or excessive healthcare costs, improper payments, or other fraudulent activities. Waste, abuse, and fraud in healthcare can result in substantial financial losses for insurance companies, which can drive up the cost of healthcare for everyone. To combat FWA in healthcare, insurance companies, regulators, and law enforcement agencies work to detect and prevent these activities, investigate potential cases, and prosecute those responsible for engaging in fraudulent activities. Fraud in Healthcare occurs when individuals or organizations intentionally deceive healthcare providers, insurance companies, or patients in order to gain some type of financial benefit. This can take many forms, including billing for services that were never performed, submitting false claims, and using someone else’s insurance information. These actions can result in improper payment or financial gain for the individuals or organizations involved, and can ultimately increase healthcare costs for patients and insurance providers. Healthcare fraud is a serious crime and can result in civil and criminal penalties, including fines, imprisonment, and exclusion from government healthcare programs.

Waste in healthcare is a significant problem that can lead to unnecessary costs without providing any or way less benefit to patients. It refers to the overuse or misuse of healthcare resources, which can result in inefficient healthcare practices and poor patient outcomes. Examples of waste in healthcare includes ordering unnecessary tests or procedures, prescribing expensive brand-name drugs when generic alternatives are available, and using higher-cost facilities for routine care. Such wastes in healthcare can contribute to rising healthcare costs and reduced access to care for patients. Addressing waste in healthcare is also important for improving the efficiency and effectiveness of healthcare delivery, while also ensuring that patients receive the appropriate care they need. This can involve implementing strategies to reduce unnecessary testing and procedures, promoting the use of cost-effective medications, and encouraging the use of lower-cost healthcare facilities for routine care.

Abuse in healthcare is another challenge that can lead to unnecessary costs and improper payments. It refers to actions that are inconsistent with accepted business or medical practices, and can result in fraudulent or unethical behavior by healthcare providers or organizations. Some of the many examples of abuse in healthcare are over-billing for services or billing for services that were not actually provided. It can lead to fraudulent or unethical behavior, resulting in unnecessary costs and improper payments. Addressing abuse in healthcare is important for improving the integrity of healthcare delivery, while also ensuring that patients receive the appropriate care they need. This can involve implementing strategies to detect and prevent abuse, such as conducting regular audits of billing practices, implementing fraud detection software, or establishing clear policies and procedures for billing and reimbursement. Healthcare billing processes are often complex, involving numerous codes, regulations, and billing procedures. Fraud detection systems must navigate this complexity to identify irregularities accurately. In addition, the unavailability of labelled data in the domain poses another challenge. Therefore, there is a need to design and develop effective unsupervised learning-based technique that can help detect and prevent health insurance fraud, provide actionable insights to relevant stakeholders, such as hospital and insurance providers.

Following are the key contributions of the research

- Use of unique user behavior patterns at transaction level
- Propose of detecting health insurance fraud by considering the interactions of multiple players, including patients, service providers, and physicians
- Propose a novel method that combines unsupervised rule mining and unsupervised classification approaches to identify fraudalent transactions
- Define a cost-based evaluation metric as compared to error-based metric that aligns well with proposed methodology

## Related work

Significant research exists related to the general insurance fraud detection in the past that focuses on data mining and machine learning techniques [16]. Researchers have mostly focused on one of the stakeholders of insurance triangle , more frequently, on the frauds done by patients or by the hospitals. In this research, all stakeholders in insurance triangle are focused for the better identification of frauds committed across the board. A summarised view of methods and techniques used for fraud or anomaly detection in healthcare as well as other domains with significant results, is presented here.

### Association rule mining

Association rule mining is not yet widely researched in the area of healthcare insurance or for any other fraudulent activities. Although it is a widely used data mining technique but still carrying some drawbacks, Yadav et al. discussed some techniques that can help improve the algorithm [17]. Saba et al. shared the initial stage of the study, by using the association rule followed by the SVM classification algorithm, they believe their model can address the discrepancies and thus reduce fraud in health insurance [18]. Sornalakshmi et al. presented the new technique by combining the MapReduce and Apriori association rule mining. MapReduce makes parallel computing very easy. However, the author believes Apriori algorithm needs to be fully implemented, as there is a lot of improvement needed in Apriori algorithms for parallel and distributed terms [19]. Authors of [20] used the algorithm in medical billing also believes that Apriori algorithm is good for finding frequent item-sets from billing database.

### Unsupervised machine learning

Data mining helps detect and prevent insurance fraud. Anomaly detection, Clustering, and classification can detect fraudulent insurance claims [21]. After finding anomalous claims, further investigation can be required to narrow the focus and identify fraud patterns. A recent research [22] highlights the current and furite chanlenges of anomaly detection Kirlidogab and Asukb [23] used longitudinal data of nine years but also suggest one-year analyses which can be beneficial for detecting “hit and run” frauds that are hard to detect over long periods. Gao [24] proposed the SSlsomap activity clustering method, SimLOF outlier detection method, and the Dempster-Shafer Theory-based evidence aggregation method to detect the unusual categories and frequencies of behaviours simultaneously. Alwan [25] shows how combining machine learning techniques with existing methods for detecting fraud can make it easier to find fraud. Specifically, the paper examines the effectiveness of several data mining techniques, including Decision Tree, Support Vector Machine, K-Nearest Neighbor, and Hidden Markov Model, in detecting credit card fraud. The findings highlight the potential of a hybrid approach that integrates these methods to enhance fraud detection.

Shang [26] suggested the use of One Class Support Vector Machine (OCSVM) for the intrusion detection. Authors describe that OCSVM in anomalies detection fields have advantages, such as fast and strong generalization ability, the less support vector, the simple model, and the great practical value [27].Recently authors of the work [28] proposed utilization of one class svm for the defect identification in railway track geometry. Maglaras [29] combined the ensemble methods and social networking metrics for the enhancement of the OCSVM, but it needs the improvement in order to decrease false positive and increase detection accuracy. Maglaras [30] developed using an OCSVM classifier and recursive k-means clustering. It is trained offline using network traces, and only severe alerts are communicated to the system. The module is part of an IDS system developed under CoCkpitCI, and its performance is stable and not affected by the selection of parameters $\nu$ and $\sigma$. However, the author believes further evaluation is needed to determine its effectiveness under different anomalies scenarios. Wang [31] proposes an improved particle swarm optimization algorithm to enhance the accuracy of the OCSVM-based power system anomaly detection. The algorithm introduces an adaptive learning factor and splitting and elimination mechanism to improve the population’s diversity and fine searching ability. Amer [32] proposed SVM-based algorithms are effective for unsupervised anomaly detection, outperforming clustering-based methods in most cases. The proposed eta one-class SVM produces the most promising results, with a sparse solution and superior AUC performance. The introduced outlier score calculation method allows for ranking of outliers, providing practical value for anomaly detection applications.

In 2008, Fei Tony Liu and Zhi-Hua Zhou developed an algorithm called the Isolation Forest [33] with the purpose of finding anomalies in data. This particular algorithm makes use of binary trees in order to identify anomalies, and because of its linear time complexity and low memory requirements, it is well suited for the processing of large amounts of data. Isolation Forest algorithm’s low accuracy, execution efficiency, and generalization ability are addressed by Xu’s SAiForest data anomaly detection method [34]. SAiForest optimises the forest by selecting isolation trees with high abnormality detection and difference using simulated annealing and selective integration based on precision and difference value. Cheng [35] proposes the union of Isolation Forest and Local Outlier Factor to detect outliers in multiple datasets. The algorithm calculates each data point’s anomaly score using binary trees and prunes normal samples to find outlier candidates. The proposed method addresses Isolation Forest’s local outlier issues and reduces Local Outlier Factor’s time complexity. Ding [36] proposes an iForest-based anomaly detection framework under the sliding windows framework iForestASD, for streaming data. Four real-world data sets show that proposed method is efficient. Authors believes there is still a lot improvement required in the algorithm, such as defining the threshold and size of sliding window. Lesouple [37] introduced generalized isolation forest for anomaly detection. Although it achieved the less execution time but the false alarm rate is high. A recent work [38] utilized autoencoder methods to find fraudulent claims and found that this technique outperformed to the density based clustering methods.

Cluster Based Local Outlier Factor (CBLOF) was proposed by He et al. in 2022 [39]. It is generally used for outlier detection that considers a combination of local distances to nearby clusters and the size of those clusters. It identifies anomalies as data points that are located in small clusters next to a larger nearby cluster. Such outliers may not be single points but instead, small groups of isolated points. John [40] explained the workings of Local Outlier Factor and Isolation Forest and suggested its use for identification of credit card fraud with the accuracy of 97% and 76% respectively. Kanyama [41] used K-Nearest Neighbor (k-NN), CBLOF, and histogram-based outlier score (HBOS) for anomaly detection in smart water metering networks. After the experimentation, authors believes that CBLOF performs better than KNN in terms of detection rates, but KNN achieved almost zero in terms of False Positive Rate. Irfan [42] performed an experiment for the evaluation of the performance of three unsupervised outlier detection algorithm such as K-Means, LOF, and CBLOF. Authour states that the CBLOF performed better than its competitors, CBLOF was faster in terms of computational complexity. Author recommended to restart the K-Means algorithm multiple times for stable cluster results, but CBLOF may be preferable for applications where processing speed or updating clustered models in streaming data is important. In another experiment Irfan [43] applied the methodology for churn prediction in banking system and came up with the same results in favor of CBLOF. The main goal of the research in this domain is to find the most important features and data sources, such as medical records, billing details, and demographic information, for using unsupervised learning techniques to find health insurance fraud. In the proposed approach, it is intended to identify the fraudulent patterns based on interaction of three stakeholders, that is, patient, physician and service. In addition, the influence of data preprocessing approaches, like normalization, feature scaling, and missing data imputation, on the accuracy and resilience of fraud detection models is studied. Moreover, the potential of ensemble methods, combining multiple unsupervised learning models to enhance accuracy and generalization is investigated by evaluating the performance of various unsupervised learning algorithms in detecting health insurance fraud.

### Dataset in use

In terms of studies utilizing the same dataset, Table 1 describes the details on research conducted on DE-synPUF dataset described in “Dataset” section. Bauder et al. [44, 45] found that the C4.5 decision tree algorithm outperformed others in terms of Area Under the Curve (AUC) metrics, indicating its efficacy in identifying fraudulent activities. Similarly, Herland et al. [46, 47] demonstrated the effectiveness of logistic regression and gradient tree boosting, achieving commendable AUC results. Fan et al. [48] highlighted the superior performance of decision tree classifiers, especially when integrating social media data, suggesting a novel approach to enhancing fraud detection accuracy.

Ekin et al. [49] noted a direct correlation between increased class imbalance and decreased AUC scores, yet they identified Random Walk Oversampling (RWO) as a potent method to counteract this issue. Sadiq et al. (2017) [50] employed a PRIM-based bump-hunting technique, effectively pinpointing potential fraudulent activities, while Sadiq et al. (2019) [51] used propensity matching and clustering in their CPM Fraud Miner to detect data anomalies indicative of fraud.

Each study, while advancing the understanding of fraud detection mechanisms, encountered limitations. These ranged from the challenges of dealing with highly imbalanced datasets, as in the case of Bauder et al., to the complexities of integrating diverse data sources, such as social media and public records, which could introduce biases into the analysis, as noted by Fan et al. Moreover, the methodologies often relied on assumptions or incomplete data, with the true extent of fraudulent activities remaining partially uncovered, thus highlighting the necessity for more comprehensive and robust data analysis techniques in future research.

For the research studies using supervised learning techniques, a key aspect is distinguishing between fraudulent and legitimate providers by identifying relevant features. While, the approach to identifying these features differs among researchers, a common predominant focus is on the provider level as opposed to the transaction level. In addition, when defining ground truth for supervised learning, the accuracy of the labeled dataset is crucial, as it directly impacts the categorization of data into specific classes. In most studies on the dataset, fraud labels are obtained by incorporating exclusions from the Office of Inspector General’s (OIG) List of Excluded Individuals/Entities (LEIE) database [54]. While the LEIE database lists provider-level exclusions, it does not comprehensively capture all instances of provider fraud. Notably, 38% of providers with fraud convictions remain in medical practice, and 21% have not been suspended from practicing medicine despite their convictions, as highlighted in Pande and Maas [55]. The integrity of provider classification into fraudulent or legitimate (non-fraudulent) is essential, yet there also remains an ambiguity for providers not previously scrutinized for fraud. Some studies have attempted to mitigate this uncertainty by estimating a range for the class distribution of unreviewed providers, highlighting the potential misclassification of fraud cases as non-fraudulent. The binary classification system, categorizing providers simply as fraudulent or legitimate, may not fully capture the amount of fraud commitment. Therefore, assessing the level of “fraud confidence” could offer a better approach for training models. In our study we evaluate our rules based on confidence and support for rule approach. Furthermore, the fraud dataset typically exhibits a skew, with a disproportionate number of providers classified as legitimate compared to those deemed fraudulent. This imbalance, known as “class imbalance,” reflects a common challenge in the dataset’s label distribution.

While unsupervised methods used are more practical, in approaches like outlier detection, the responsibility to establish fraudulent intent falls to investigators or experts. Identifying specific claim line details that underpin the fraud is also challenging, given that such billing discrepancies often pertain to the overall behavior of the provider. Therefore, in our study we employ the unsupervised methods in combination with rule-based approach for detection to mitigate some shortcomings of unsupervised approaches.

**Table 1:** Study Methods and Features for Work on DE-synPUF dataset

Study reference	Labeling approach	Techniques used
Bauder et al. [44, 45]	LEIE	Random Forest, C4.5, SVM, Logistic Regression
Herland et al. [46, 47]	LEIE	Logistic Regression, Gradient Tree Boosting
Fan et al. [48]	LEIE	Logistic Regression, Naïve Bayes, Decision Tree
Ekin et al. [49]	Unsupervised	PCA, RWO, co-clustering
Sadiq et al. [50] (2017)	PRIM	bump hunting
Sadiq et al. [51] (2019)	unsupervised	Cascaded Propensity Matching (CPM) Fraud Miner
Zafari and Ekin [52]	Unsupervised	Topic modeling, outlier detection
Ekin et al. [53] (2019)	Unsupervised	Bayesian model, Gibbs sampling

Based on the related work, a potential research area that is explored is the limited application of association rule mining for fraud detection across all stakeholders in the insurance triangle (patients, physicians, and services). Additionally, exploring the integration of association rule mining with other techniques like unsupervised classifiers or ensemble methods could further enhance the accuracy and effectiveness of fraud detection systems in the healthcare insurance domain.

## Materials & methods

### Dataset

In this study, the Centers for Medicare and Medicaid Services (CMS) Linkable 2008-2010 Medicare Data Entrepreneurs’ Synthetic Public Use File DE-synPUF is utilized (https://www.cms.gov/data-research/statistics-trends-and-reports/medicare-claims-synthetic-public-use-files/cms-2008-2010-data-entrepreneurs-synthetic-public-use-file-de-synpuf). The claims made by Medicare recipients and a random sample of five percent of those beneficiaries from 2008 to 2010 are included in the dataset. The CMS made twenty random sample files available for researchers. The inpatient dataset from subsample 1 of the available files is utilized in this study. While, there is nothing that restricts using only this one sample or using multiple samples at the same time, studies have suggested that inpatient fraud may be more prevalent than outpatient fraud. One of the possible explanation for this is that inpatient care tends to be more expensive than outpatient care, which means that there is a greater potential for fraudulent activity to generate large profits. Additionally, inpatient care may involve more complex procedure and treatments, which can be easier to over bill or manipulate as compare to simpler outpatient services. The selection of this particular method for validating the proposed methodology was completely arbitrary, and in future more samples can be added to the dataset.

**Fig. 3:**
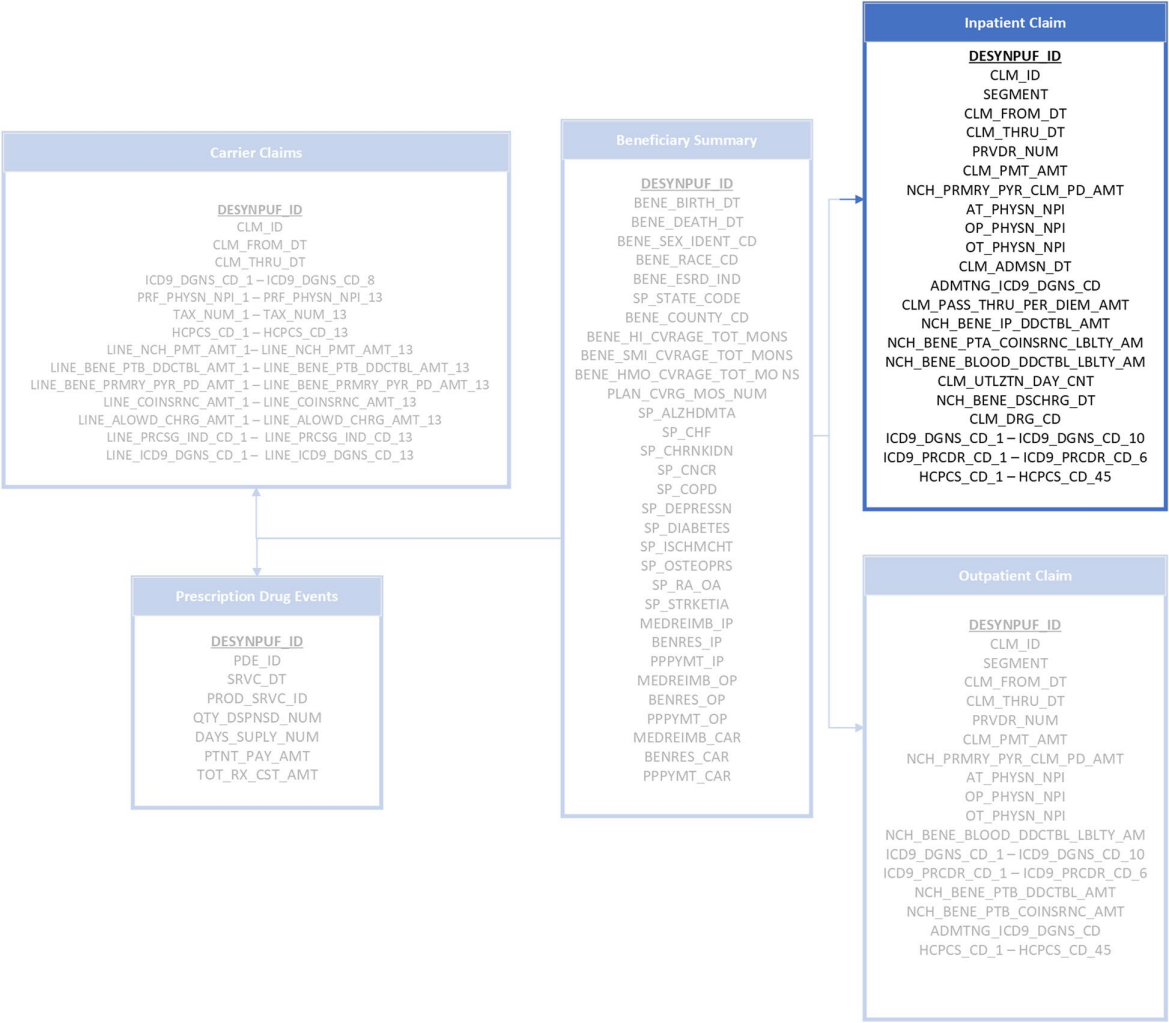
Inpatient Claim extracted from Carrier Claims, Prescription Drug Events, Beneficiary Summary and Outpatient Claim of DE-synPUF dataset

The selected sample consists of following features. The beneficiary code (DESYNPUF_ID) identifies each beneficiary in the dataset, while the claim ID distinguishes claims for the same beneficiary. A record’s claim line section identifies its claim component. The start and end dates indicate the claim period. The provider institution is the medical facility that performed the service, and the claim payment amount is the total amount paid. Attending, operating, and other physician NPI numbers identify service providers. The inpatient admission and discharge dates show when the beneficiary was hospitalised. Diagnosis and procedure codes define illnesses and treatments. Lastly, the revenue centre HCFA common procedure coding system classifies medical service.The attributes are presented graphically in the Fig. 3.

### Baseline methods

The unsupervised base-line learning techniques used in this research include Apriori, Isolation Forest, One-Class SVM i.e OCSVM, Clustering-based Local Outlier Factor (CBLOF), and Ensemble Correlation-Based Outlier Detection (ECOD). Apriori is a well-known algorithm for mining frequent itemsets and association rules, which is used to identify patterns and relationships between different items in a dataset. Isolation Forest is a tree-based algorithm that partitions the dataset into isolated subspaces, which is used to detect anomalies and outliers. OCSVM is a support vector machine-based algorithm that creates a boundary around the normal data points, which is used to identify anomalous data points that fall outside the boundary. OCSVM is relevant for anomaly detection due to its ability to identify outliers or anomalies in datasets where only one class (normal instances) is predominantly represented. CBLOF is a clustering-based approach that uses k-means clustering to identify local outlier factors, which is used to identify anomalous clusters. ECOD is an ensemble method that combines multiple correlation-based outlier detection methods, which is used to identify anomalous data points that are consistent across multiple methods.

#### Apriori algorithm

Agrawal and Srikant proposed the Apriori algorithm in 1994, which has become a widely used data mining algorithm for identifying frequent item sets in a transaction database [56]. In the field of association rule mining, the Apriori algorithm is recognized as one of the most well-known algorithms [57]. However, it may not be the optimal choice for detecting anomalies or fraudulent transactions in a database. This is because it is commonly assumed that fraudulent transactions are significantly fewer than normal ones. Therefore, when implementing Apriori, it is expected that the algorithm will generate rules based on normal transactions.

Apriori algorithm works in two steps for association rule mining. The first step is to find all the frequently occurring item sets from the data and generating association rules from the set of frequently occurring items is done in the second step [58].

#### Isolation forest

**Fig. 4:**
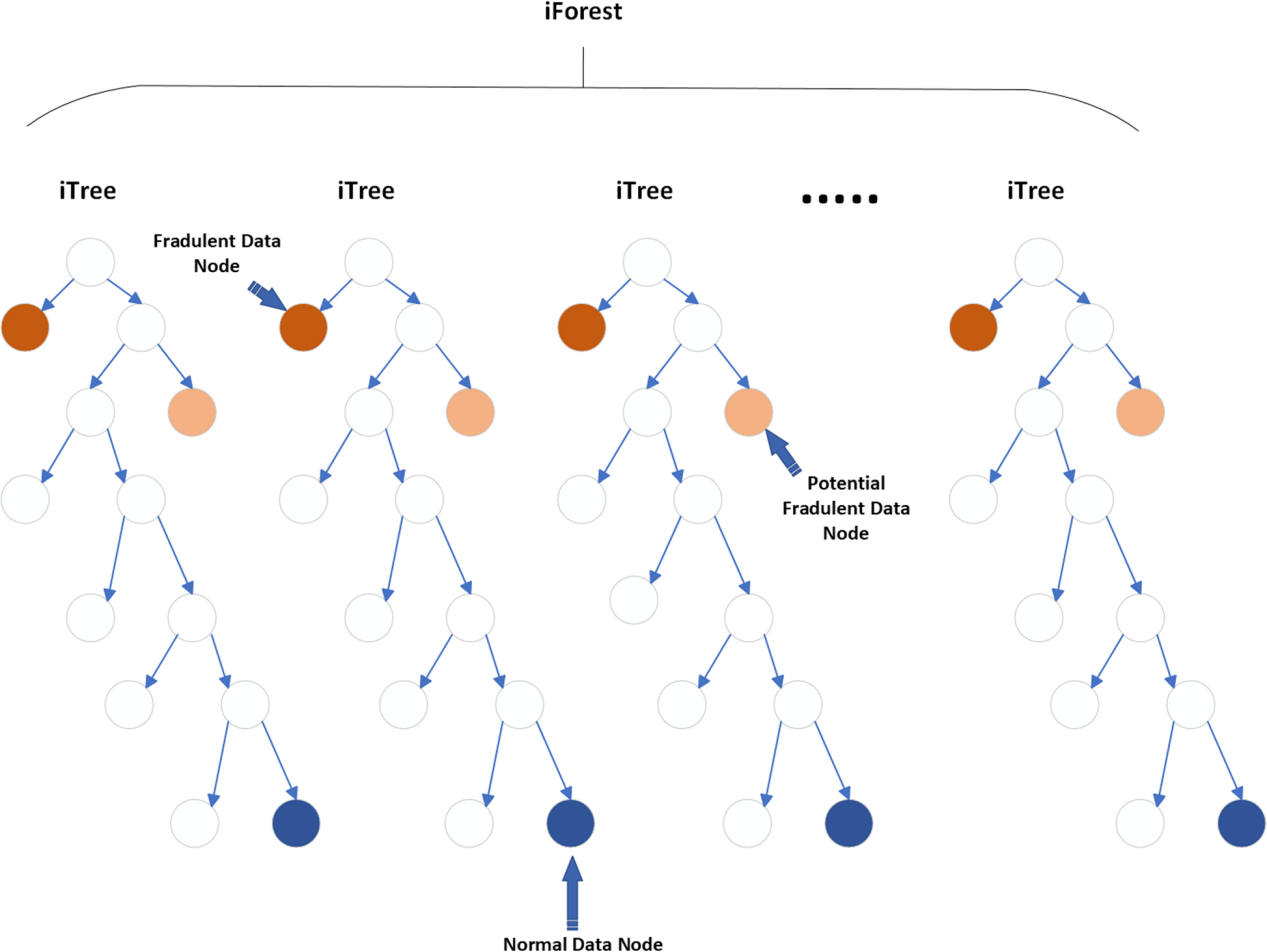
Isolation Forest

Isolation Forest was introduced at Lie et al. [33] in 2008. Generally, it is designed to detect anomalies from structured data. The iTree, or isolation tree, is a binary tree data structure in which each node corresponds to a subset of data objects. The tree is constructed by randomly sub sampling a subset of n data objects from the entire dataset and using it as the data pool for the root node. The tree grows by recursively partitioning the data objects in the leaf node into two child nodes, until a single data object remains in the node or the maximum depth limit is reached. The branching criterion for each data object is determined by comparing a randomly selected feature of the data object to a split value within the range of that feature’s values. The path length of a data object in the iTree serves as an indication of the object’s abnormal degree. An iForest, or isolation forest, is constructed by creating multiple iTrees, and the anomaly score of a data object is calculated by averaging the path lengths of that object across all iTrees in the forest. The final anomaly score is then normalized using a factor.The visual representation is shown in the Fig. 4 .

Isolation Forest consists of two steps, training and testing phase. In training, the algorithm builds an ensemble of isolation trees, known as iTreesEach tree is build through algorithm. By default 100 iTrees are built in an IForest but changes can be made in experiments for obtaining the best results.

**Figure:**
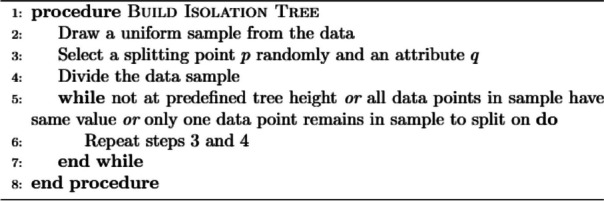
**Algorithm 1** Building a decision tree

In the next step of IF algorithm, each data point is passed through each built iTree to calculate its corresponding anomaly score *a*(*x*) from 0 to 1. Labels are assigned based on their respective data point’s scores. Specifically, those with scores below 0.5 are classified as normal and receive a label of 1. On the other hand, data points with scores that are closer to 1 are deemed as potential anomalies and thus labeled with a value of -1.

Anomalies are detected through

1 $$\begin{aligned} a(x, m) = 2 \frac{-E(h(x))}{k(m)} \end{aligned}$$

where *c*(*m*) is a normalization constant for a data set of size *n*. The expression E(h(x)) represents the expected or “average” value of this path length across all the Isolation Trees. The expression k(m) represents the average value of h(x) given a sample size of m and is defined using the following equation. Following equation illustrates the formula of the constant k(m).

2 $$\begin{aligned} c(m) = \left\{ \begin{array}{cl} 2H (m - 1) - \frac{2(m-1)}{m} &{} : \ for \ m > 2 \\ 1 &{} : \ for \ m = 2 \\ 0 &{} : \ otherwise \end{array} \right. \end{aligned}$$

where *H* is the harmonic number, which can be estimated by $H(i)=\ln (i)+\gamma$, where $\gamma =0.5772156649$ is the Euler-Mascheroni constant.

#### Cluster based local outlier factor

**Fig. 5:**
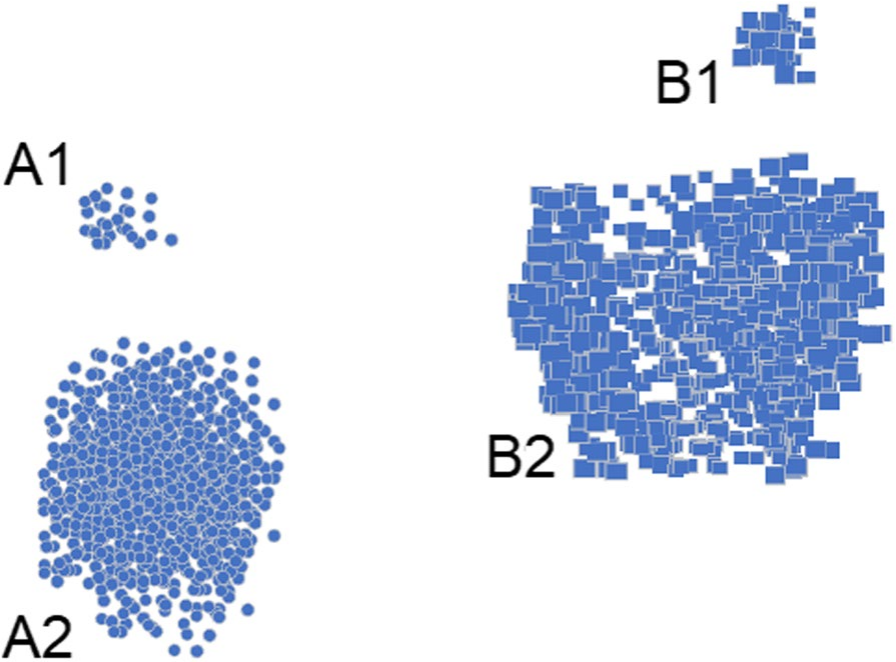
Cluster Based Local Outlier Factor

Cluster-Based Local Outlier Factor (CBLOF) was proposed by He et al. [39] in 2002. The CBLOF definition of anomalies takes into account both the local distances to neighbouring clusters as well as the sizes of the clusters to which the data point belongs. Algorithm first cluster next to a nearby large cluster are identified as outliers. The Local outliers may not be a singular point, but a small group of isolated points as shown in Fig. 5.

In general, the procedure of CBLOF can be described in the three steps. Initially, a data point is assigned to one and only one cluster. K-means is commonly used as clusteric algorithm for CBLOF. Next, CBLOF ranks clusters according to the cluster size from large to small and get the cumulative data counts. Clusters that holds 90% of the data are considered as “large” clusters rest of them are consider as “small” clusters. The threshold of 0.9 can be fine-tuned as per requirement. Lastly, the outlier detection process involves the calculation of the distance of a data point to the centroid and its corresponding outlier score. For data points belonging to a large cluster, the distance is calculated as the distance from the data point to the centroid of its cluster. The outlier score is then determined as the product of this distance and the number of data points in the cluster. For the smaller clusters the distance is the distance from the data point to the centroid of the nearest large cluster. The outlier score for these data points is determined as the product of this distance and the size of the small cluster to which the data point belongs.

As it can be seen in Fig. 3, clusters A1 and B1 are the smaller clusters and A2, and B2 are large cluster. A1 and B1 will be considered as outlier as they do not belong to any of the large clusters A2 and B2. According to the local neighborhood, data in cluster A1 is local outliers to A2, and same with B1 for B2.

#### One-class support vector machine

An unsupervised learning technique, One-Class Support Vector Machine (OCSVM) is used for outlier detection and constituting an incremental learning process. Its application in Anomaly Detection is widely used around the world such as Outlier Detection, Novelty Detection, and many others. OCSVM is modified to be a single-class learner from SVM that tries to find a hyper-sphere among the instances of the normal classes. This model classifies new data as normal or abnormal, all observations inside the hyper-sphere are normal and those outside the hyper-sphere and abnormal or anomalies.

Let us first examine the conventional two-class support vector machine. Consider a data set with two dimensional space $(x_1,y_1),(x_2,y_2),\dots ,(x_n,y_n)$ $;$ points $x_i \in \mathbb {R}^d$ where $x_i$ is the *i*-th input data point and $y_i \in \{-1,1\}$ is the *i*-th output pattern, indicating the class membership.

A significant advantage of support vector machines (SVMs) is their capability of generating a non-linear decision boundary by transforming the data through a non-linear mapping $\phi$ to a higher-dimensional feature space *F*. In this feature space, it may be possible to separate the classes with a hyperplane, even if a linear boundary is not feasible in the original input space *I*. This process results in a non-linear curve in the input space when the hyperplane is projected back. By utilizing a polynomial kernel for the projection, all the dots are elevated to the third dimension, and a hyperplane can be employed for separation. When the plane’s intersection with the space is projected back to the two-dimensional space, it results in a circular boundary.

The hyperplane that separates the classes in an SVM is represented by the equation $w^Tx + b = 0$, where *w* is a vector in the feature space *F* and *b* is a scalar in $\mathbb {R}$. The margin between the classes is determined by this hyperplane, with all data points belonging to class $-1$ on one side and all data points belonging to class 1 on the other. The hyperplane aims to maximize the distance between the closest data points from each class to itself, thus achieving the maximum margin or “separating power.”

To address the issue of overfitting in the presence of noisy data, slack variables $\xi _i$ are introduced to permit some data points to lie within the margin. The trade-off between maximizing the margin and accommodating training errors is controlled by the constant $C > 0$. The SVM classifier’s objective function is a minimization formulation that balances these factors.

3 $$\begin{aligned} \min _{w,b,\xi _i} \ \frac{1}{2}\Vert w\Vert ^2 + C\sum \limits _{i=1}^n\xi _i \end{aligned}$$

$$\begin{aligned} \begin{array}{c} \ \text {subject to: } \\ y_i\left(w^T\phi (x_i) + b\right) \ge 1-\xi _i \quad \text {where } i =1,\dots ,n\\ \xi _i \ge 0 \quad \text {where } i =1,\dots ,n \end{array} \end{aligned}$$

According to Scholkopf et al. [59], separates all the data points from the origin in the feature space *F* and maximizes the distance from hyperplane to the origin. This result in a binary function which returns +1 in a “smaller” region and -1 elsewhere.

4 $$\begin{aligned} \min _{w,\xi _i,\rho } \frac{1}{2}\Vert w\Vert ^2 + \frac{1}{\nu n} \sum \limits _{i=1}^{n} \xi _i - \rho \ \end{aligned}$$

$$\begin{aligned} \begin{array}{c} \text {subject to:} \\ (w \cdot \phi (x_i)) \ge \rho - \xi _i \quad \text {where } i =1,\dots ,n\\ \xi _i \ge 0 \quad \text {for } i =1,\dots ,n \end{array} \end{aligned}$$

By using Lagrange techniques and using a kernel function for the dot product calculations, the decision function becomes:

5 $$\begin{aligned} f(x) = \text {sgn}((w \cdot \phi (x))-\rho ) = \text {sgn}\left( \sum \limits _{i=1}^n \alpha _i K(x,x_i) - \rho \right) \end{aligned}$$

#### Empirical cumulative distribution based outlier detection

The Empirical Cumulative Distribution-based Outlier Detection (ECOD) method has several advantageous attributes that distinguish it from alternative algorithms. ECOD is unique in its lack of dependence on hyperparameters, its computational efficiency and swiftness, and its ease of interpretation and comprehension. The ECOD approach leverages information regarding the distribution of data to identify points that deviate significantly from the majority, thus indicating their outlier status. The ECOD technique calculates the tail probability of each variable using univariate Empirical Cumulative Distribution Functions $\left( \delta \right)$ and combines these probabilities through multiplication.

Detection of the anomalies through ECOD is done through the computation of three values. ECDfs are used to generate the left- and right-tail probability values,

1. *O-left = Sum of the negative log of the left-tail probability of every variable*
2. *O-right = Sum of the negative log of the right-tail probability of every variable*
3. *O-auto = Sum of left- or right-tail probability of every variable, depending on whether it is left- or right skewed*

Final outlier score of an observation is obtained through taking the extreme negative log probability score.

6 $$\begin{aligned} \textit{Outlier Score} = max(O_{left}, O_{right}, O_{auto}) \end{aligned}$$

For mathematically-inclined, following are simplified formulations of the three equations describe above

7 $$\begin{aligned} O_{left} = -\sum \limits _{j=1}^{d}\log \left( \delta ^j_{left}\left( X^j\right) \right) \end{aligned}$$

8 $$\begin{aligned} O_{right} = -\sum \limits _{j=1}^{d}\log \left( \delta ^j_{right}\left( X^j\right) \right) \end{aligned}$$

9 $$\begin{aligned} O_{auto} = -\sum \limits _{j=1}^{d} \Biggl \{ \begin{array}{cl} \log \left( \delta ^j_{left}\left( X^j\right) \right) &{} if \gamma _j < 0 \\ \log \left( \delta ^j_{right}\left( X^j\right) \right) &{} if \gamma _j \ge 0 \end{array} \end{aligned}$$

*where* $\gamma _j$ *is the skewness coefficient*

### Proposed methodology

The proposed methodology is designed based on two features of healthcare ecosystem. First, since multiple entities are involved in a health insurance claim including service provider, beneficiary, service and claim, it is important to analyze a transaction in in context of interactions among these entities. Second, rules provide context by showing how various factors interact in an ecosystem, consequently also show the expected behaviour of the system. Based on these two characteristics of the health insurance claims, we use features from three entities, including patient, provider and physician that are represented in the claims data where each instance is a transaction with items corresponding to features of the three players. Apriori is used to mine association rules in the claims transactional data between features. These association rules indicate which features tend to co-occur frequently in instances. Features that are part of strong association rules are considered important or informative. Based on the association rules generated by Apriori, we filter out features that do not meet the support and confidence thresholds. Features that are part of strong association rules with high support and confidence values are retained as selected features.

**Fig. 6:**
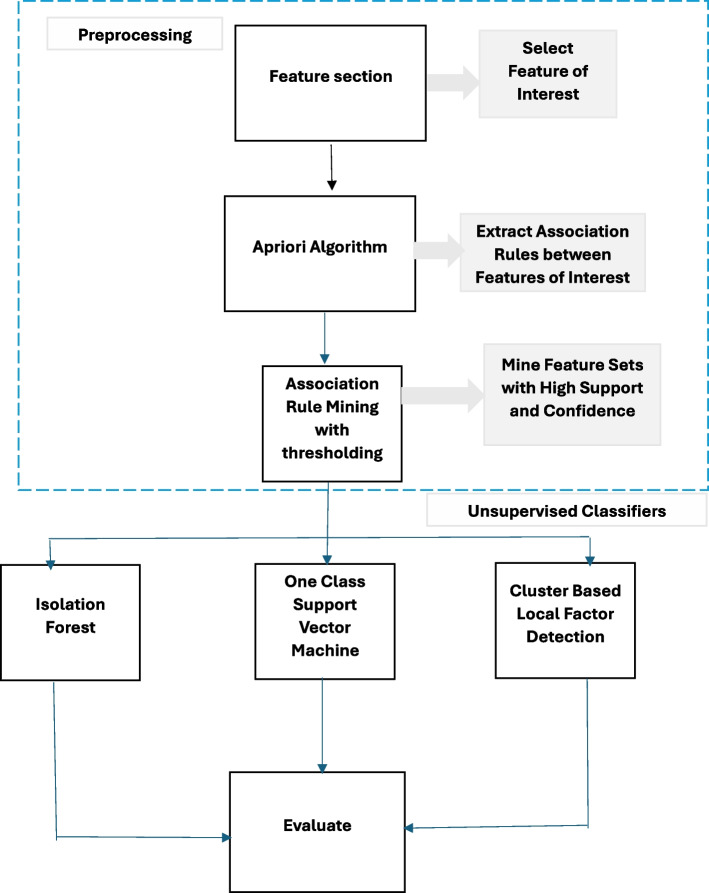
Proposed Ensemble Methodology

Rules capture patterns and associations in the data that may not be evident when analyzing individual transactions. By identifying fraudulent rules from the set of all extracted, a broader understanding of how fraudsters manipulate the system is gained, potentially uncovering more comprehensive fraudulent schemes. Focusing on individual transactions can lead to a high rate of false positives, where legitimate transactions are wrongly flagged as fraudulent due to isolated anomalies. Identifying fraudulent rules allows for a more nuanced approach, reducing false alarms by considering patterns over multiple transactions. Fraudsters continuously evolve their tactics. Identifying rules allows your fraud detection system to adapt to new fraud schemes by detecting changes in patterns and associations, even if the specific transactions involved differ.

**Fig. 7:**
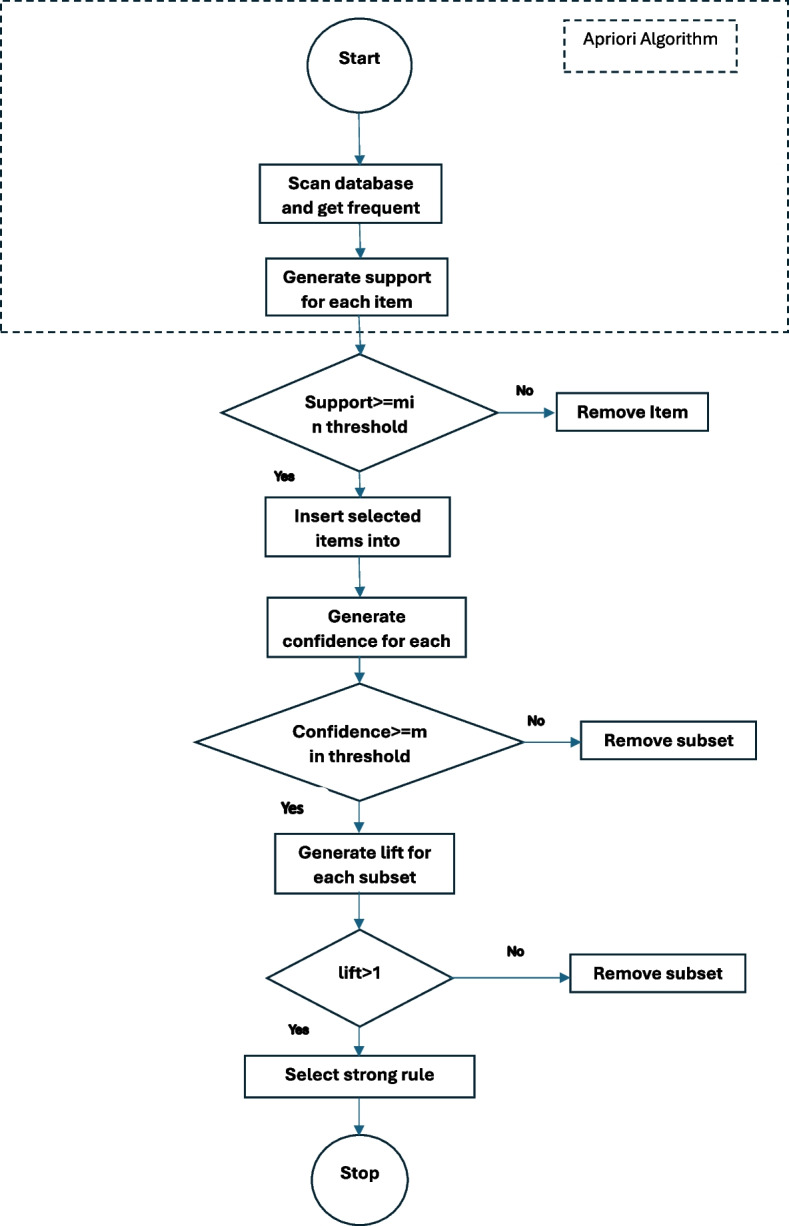
Association Rule Mining Process Flow Chart

A methodology is proposed reference to the Fig. 6 that initiates with the Apriori association rule mining algorithm to derive a set of rules. During association rule mining as shown in the Fig. 7 , it is imperative to apply filters to the mined association rules employing statistical metrics such as support, confidence, and lift ratio. Support denotes the frequency with which an association rule manifests in the dataset, while confidence quantifies the reliability of a rule’s computation. The lift ratio gauges the strength of the association between the antecedents and consequences of the rule. Hence, association rules with support, confidence, and lift ratios falling below predefined thresholds are considered as potential candidates for fraudulent rules. Subsequently, a classifier is employed to categorize these identified rules into fraudulent or non-fraudulent categories, utilizing unsupervised methodologies applied to the Apriori-generated rules.

In essence, our methodology integrates rule mining, statistical filtering, and machine learning-based classification to identify and distinguish potentially fraudulent rules within the dataset. This approach allows for a more refined and data-driven assessment of suspicious patterns, enhancing the efficiency and accuracy of fraud detection in complex healthcare insurance transactions.

### Evaluation metrics

The traditional methods used on the dataset for evaluation are “error-based” focusing primarily on minimizing the number and severity of mistakes in fraud prediction, such as false positives and false negatives. However, this approach is limited in its ability to impact the financial and operational implications of fraud detection efforts. The main problem with the error-based approaches is that it does not account for the varying costs associated with different types of errors. For example, the cost of a false positive (wrongly flagging a legitimate claim as fraudulent) can be vastly different from that of a false negative (failing to detect an actual fraudulent claim). In a healthcare insurance context, the latter might lead to substantial financial losses and undermine the integrity of the insurance system.

Given these challenges, a cost-based evaluation metric becomes more applicable and relevant. This approach incorporates the financial impact of fraud detection decisions, prioritizing actions that save the most money or resources for the insurance provider. Coverage-based metrics align well with the cost-based approach in this context. Coverage reflects the proportion of fraudulent activities that the detection system can identify across the dataset. A high coverage rate means that the system can effectively identify a large portion of fraudulent claims, which is crucial for minimizing financial losses in health insurance fraud. This metric complements the cost-based approach by ensuring that the fraud detection efforts are not just accurate in terms of error minimization but are also comprehensive and financially prudent, addressing the most costly or impactful fraudulent activities first.

For aligning the coverage based metrics to our association rule mining algorithm, we use support, confidence, lift and leverage given in equations 10-13 that evaluate the quality of the resulted rules separately. For Apriori algorithm, support refers to the frequency of an itemset in the database, while confidence measure the strength of the association between two itemsets. We then define cover for each rule that captures the different dependencies between the rules based on the coverage criteria given by given by 14.

10 $$\begin{aligned} support(A \rightarrow B) = P(A \cup B) \end{aligned}$$

11 $$\begin{aligned} confidence(A \rightarrow B) = P(B/A) = \frac{P(A \cup B)}{P(A) \times P(B)} \end{aligned}$$

12 $$\begin{aligned} lift(A \rightarrow B) = \frac{confidence(A \rightarrow B)}{support (B)} \end{aligned}$$

13 $$\begin{aligned} \begin{array}{c} leverage(A \rightarrow B) = support(A \rightarrow B) - (support(A) \times support(B))\\ Where \ A\ and\ B\ are\ the\ itemsets\ occuring\ in\ the\ database. \end{array} \end{aligned}$$

14 $$\begin{aligned} Cover(Rule) = \frac{1}{k} \sum \limits _{r_j \in R, i \ne j} Distance(r_i, r_j) \end{aligned}$$

Here, *Cover*(*Rule*) measures the average distance of every rule with every other rule.

In order to evaluate the results of unsupervised techniques including Isolation Forest, CBLOF, ECOD, and OCSVM, there are a variety of validity metrics proposed where most popular is Silhouette Score [60]. The silhouette coefficient is calculated by taking into account the mean intra-cluster distance *a* and the mean nearest-cluster distance *b* for each data point i.e. $(b - a)/max(a, b)$ [61]. A silhouette score near +1 indicates correct cluster, near 0 suggests possible alternative cluster, and near -1 indicates wrong cluster.

## Results and discussion

This section presents the results and discussion of research on healthcare insurance fraud detection using data mining techniques. The study utilized the open-source CMS 2008-2010 DE-SynPUF dataset, which was preprocessed by removing less important features and encoding the data.

### Descriptive analysis

The descriptive analysis allows to identify patterns, trends, and relationships in the data, which assists in drawing important conclusions and making informed decisions. The dataset consists of 66,773 insurance claim records. To streamline the analysis, features related to the Health Care Common Procedure Coding System (HCPCS) are excluded. These codes represent procedures, supplies, products, and services that may be provided to Medicare beneficiaries and individuals enrolled in private health insurance programs. By removing these features, the most relevant and informative features in the inpatient dataset is key focus here.

**Fig. 8:**
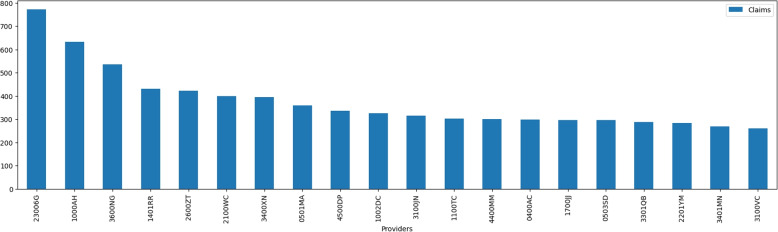
Provider Institutions Occurrence in Entire Dataset

As shown in Fig. 8, the dataset contains 2675 unique provider institutions, with 50% of the total occurring less than 10 times in the complete dataset. The provider institution “23006G” occurred in 772 records. The 20 most-occurring institution providers share the count of 7524 transactions. 209 provider institutions were only seen once in the complete dataset. The dataset contains a large number of unique provider institutions, but the majority of these institutions occur very few times in the dataset. Additionally, there are a small number of provider institutions that occur frequently, with the top 20 accounting for a significant proportion of the transactions. Finally, a substantial proportion of the provider institutions in the dataset are only seen once. This information can be used to inform further analysis of the dataset, such as identifying outliers or patterns in the data.

**Fig. 9:**
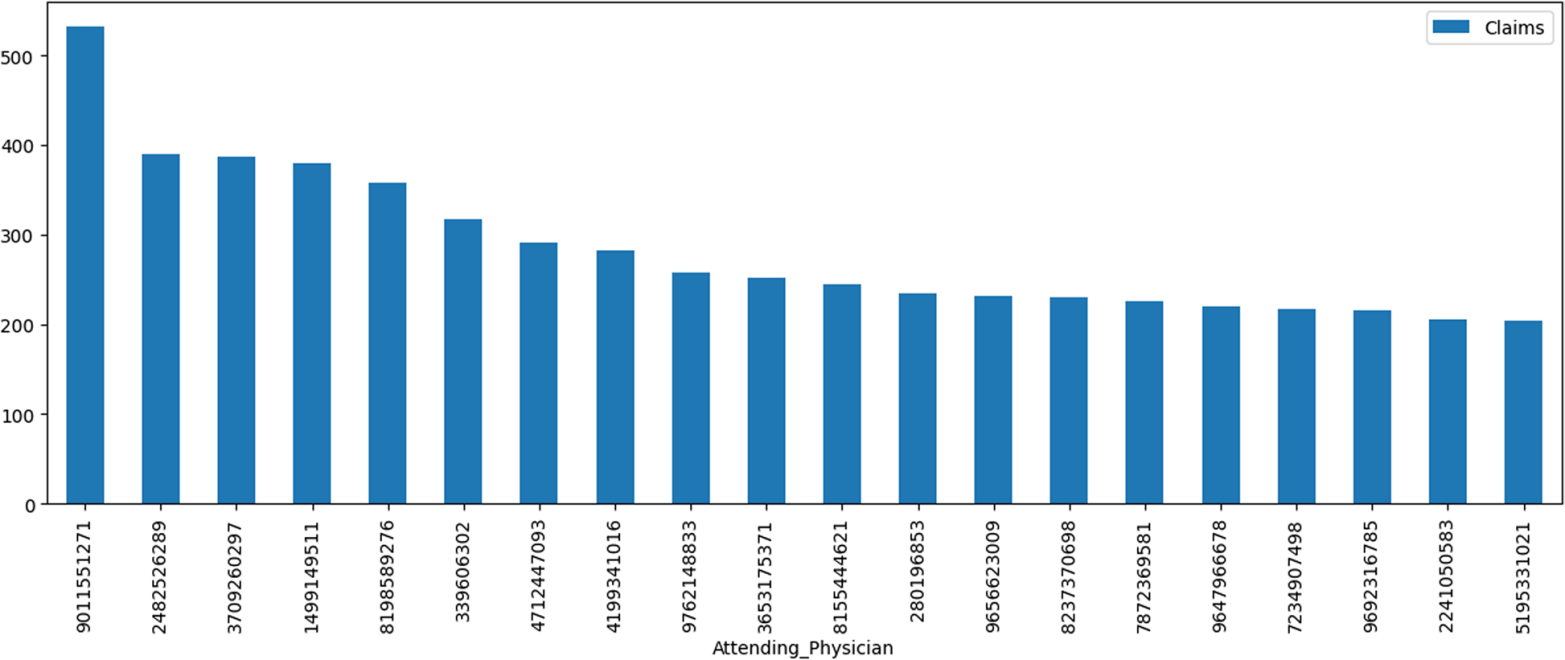
Attending Physicians Occurrences in Entire Dataset

The Fig. 9 shows 16670 unique attending physicians in the dataset while 75% of the physicians appear only once or twice. Attending Physician with id ‘9011551271’ appears in 533 transactions. The 20 most appearing attending physician share 5675 transactions. This information suggests that there is a large degree of variation in the frequency of attending physicians in the DE-synPUF dataset. While a small number of physicians occur frequently, the majority occur infrequently, which may have implications for analysis of the data.

**Fig. 10:**
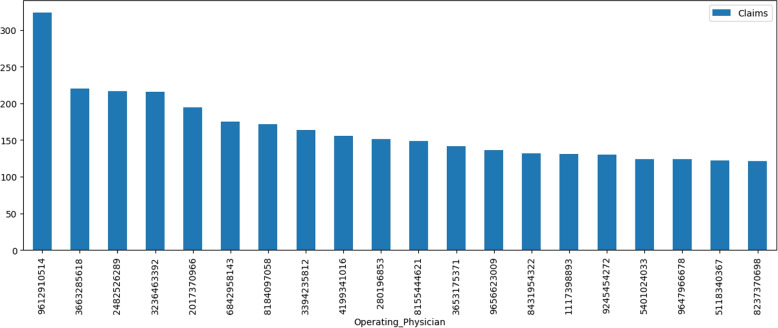
Operating Physicians Occurrences in Entire Dataset

Figure 10 shows the occurrence of top 20 operating physicians. The term operating physician refers to a physician (e.g., surgeon) who performs an operative procedure in the medical centre and who has the responsibilities outlined in the medical staff rules and regulations. The dataset contains 12076 unique operating physicians, while 75% of the physicians appear only once or twice. The operating physician with id ‘9612910514’ appears 324 times which is the highest occurrence. The 30 most frequent operating physicians shared 4377 transactions. This information suggests that there is a large degree of variation in the frequency of operating physicians in the dataset. While a small number of physicians occur frequently, the majority occur infrequently, which may have implications for analysis of the data.

**Fig. 11:**
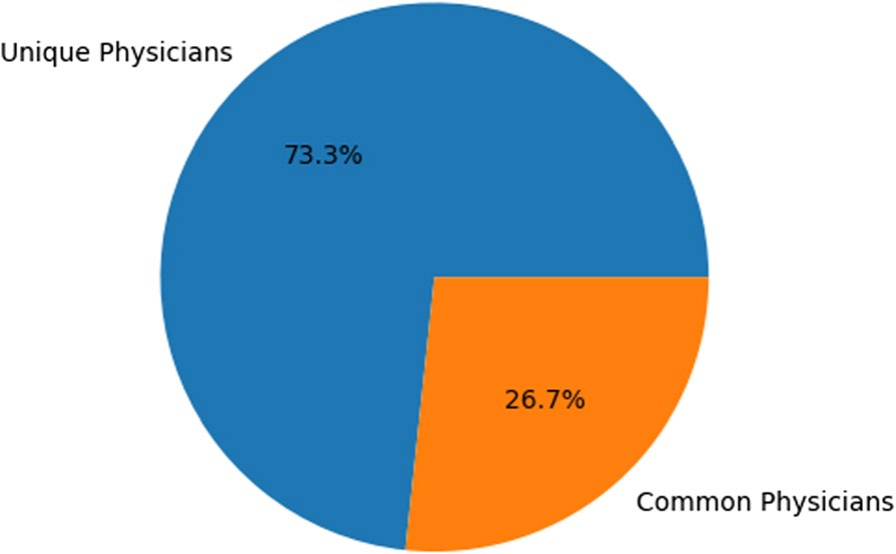
Unique & Common Physicians in Attending Physicians and Operating Physicians

Upon comparing the features of attending physicians and operating physicians, it can be seen in Fig. 11 that 26.7% of the physicians were found in both features.

**Fig. 12:**
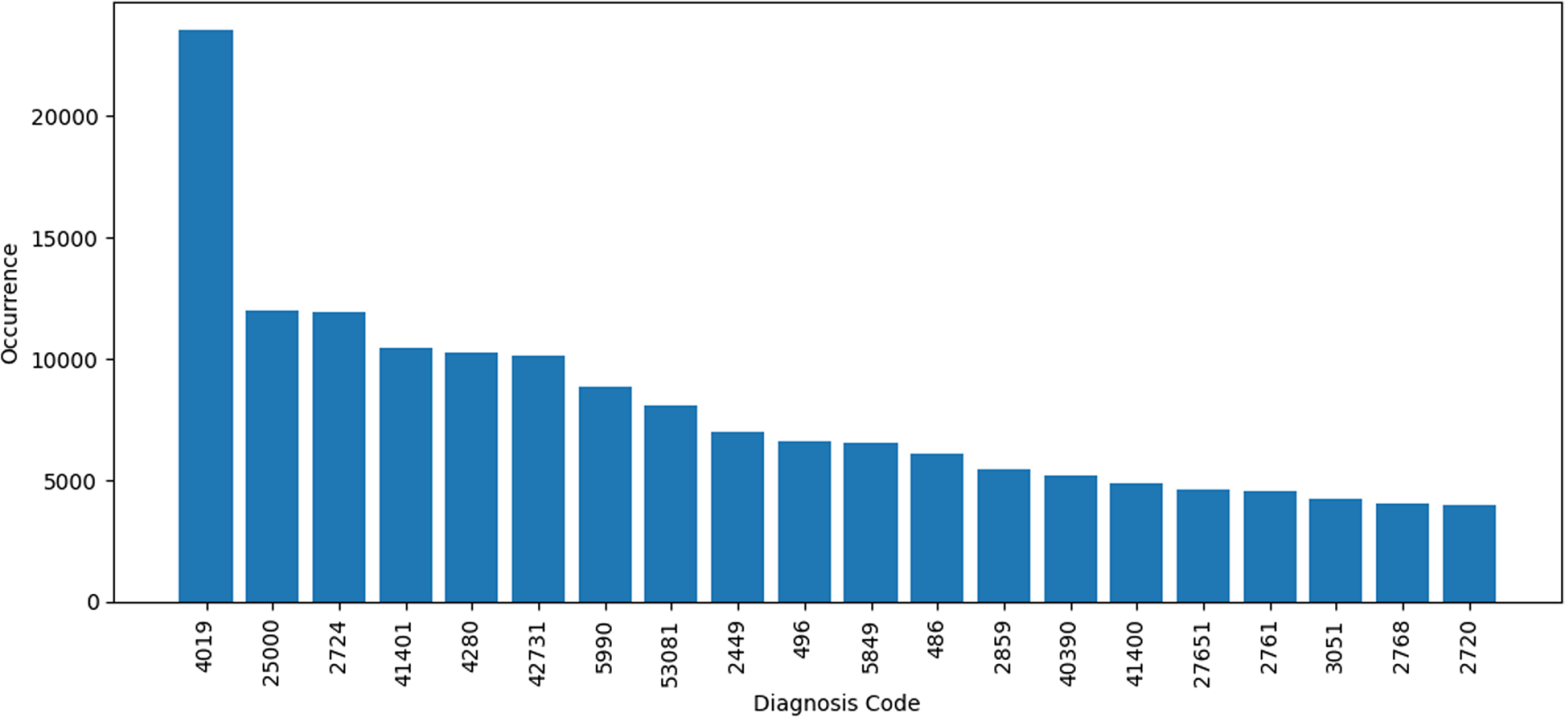
All Diagnosis Codes With Occurrences in the Dataset, 4019 Occurred the Most

In terms of features related to diagnosis codes, the dataset contains 5357 unique diagnosis codes. 50% of the diagnosis codes appear in fewer than seven transactions. The diagnosis codes are present under the mapping of ICD-9 coding. Diagnosis code ‘4019’ appears 23512 times, and referred to hypertension. Hypertension is also known as high blood pressure. The second most frequently occurring diagnosis code is ‘25000’ which is commonly known as diabetes mellitus without mention of complication, type II or unspecified type, not stated as uncontrolled. Figure 12 refers to the 20 most frequent diagnoses in the transactions.

**Fig. 13:**
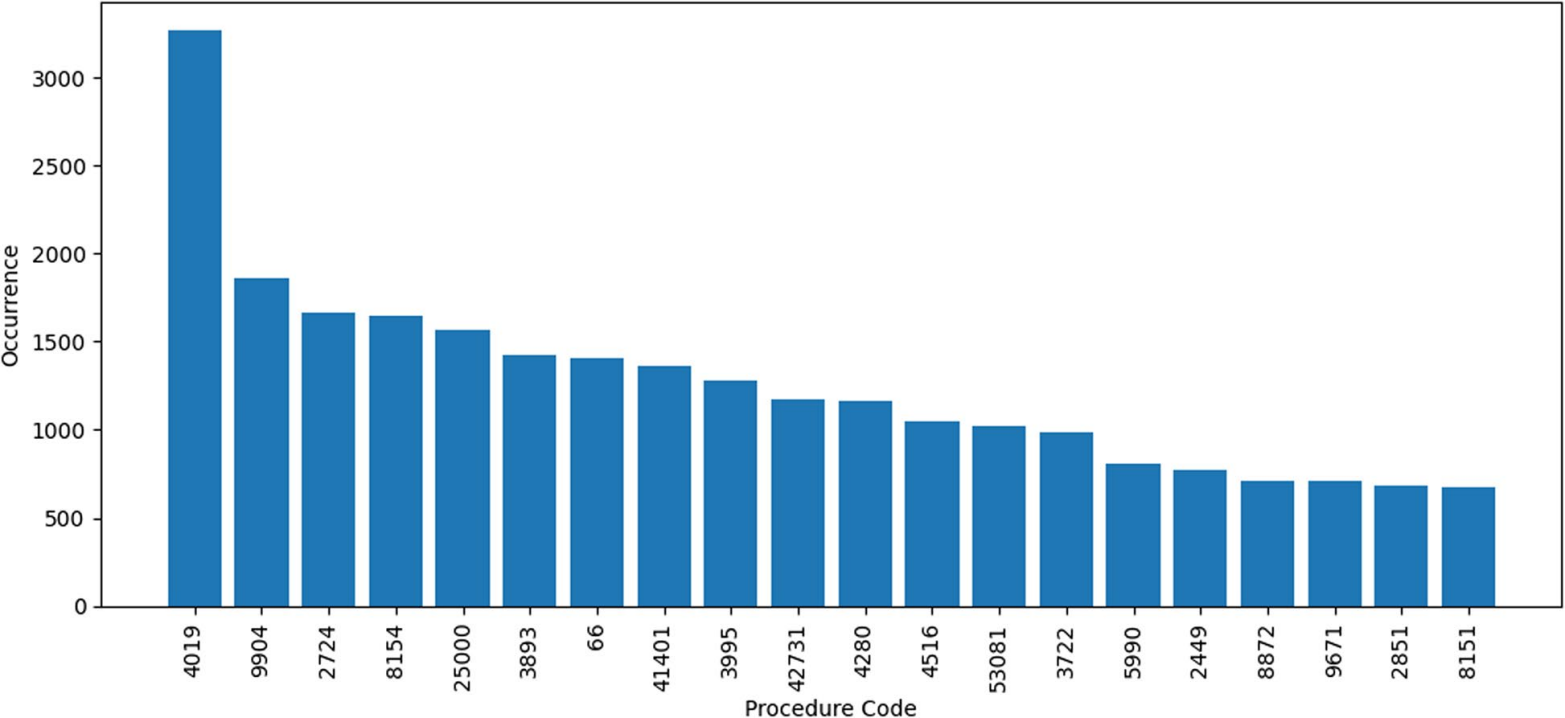
All Procedure Codes with Occurrences in Dataset

The procedure codes are also compared to the diagnosis codes. In some transactions, the procedure codes in Fig. 13 are the same as the diagnosis codes, Fig. 12. A detailed breakdown of the results of this analysis can be found in the Table 2. Except the feature procedure code 1, all of the other features has up to 35% same codes as diagnosis codes.

**Table 2:** Procedure Codes Similarity Check W.R.T. Diagnosis Codes

Feature	Unique %	Common %
Procedure Code 1	95.4%	4.6%
Procedure Code 2	64.2%	35.8%
Procedure Code 3	69.6%	30.4%
Procedure Code 4	75%	25%
Procedure Code 5	79.3%	20.7%
Procedure Code 6	83.0%	17.0%

**Fig. 14:**
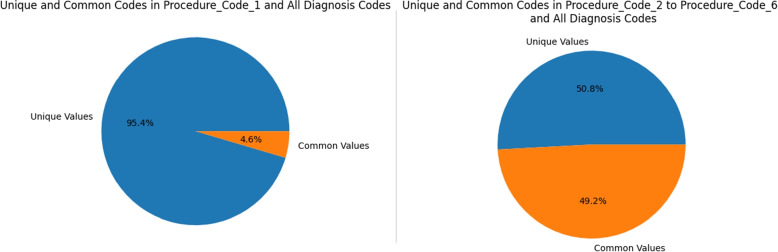
Codes Comparison Summary

Figure 14 can be referred as overall summary overall summary of finding the common codes between diagnosis and procedure codes. Feature Procedure_Code_1 has more than 95% of the procedure codes and rest of the features only have around 50% and also contains diagnosis codes.

### Preprocessing

Healthcare insurance fraud is widespread problem and can be perpetrated though various means, including upcoding, misrepresenting procedures to obtain payment for non-covered services, over billing, waiving patient copays or deductibles, and forging or altering medical bills or receipts. Identity theft is also a common way to commit health insurance fraud [62, 63]. Insurance fraud are often performed through the partnership of the services provider, patients, and hospital. Fraudster play through the technicalities of billing, unnecessary treatments and unnecessary procedures in order to get unjust benefits.

For this study, nine features are identified based on their relevance to the research question and their potential to provide insight into the relationships or patterns of interest. For example, $Provider_Institution$ is relevant for understanding the quality of care provided to beneficiaries, while $Claim_Payment_Amount$ is important for investigating the financial implications of Medicare claims. $Attending_Physician$, $Operating_Physician$, and $Other_Physician$ are useful in identifying patterns of physician involvement in care, while $Claim_Admitting_Diagnosis_Code$, $Claim_Day_Spent$, $Claim_Diagnosis_Related_Group_Code$, and $Claim_Procedure_Code_1$ provide insight into the types of medical conditions and procedures that are most common among Medicare beneficiaries. Ultimately, the selection of these features is based on their potential to answer the research question.

**Table 3:** Description of features chosen from the DE-SynPUF

Features	Description	Type
Provider_Institution	Unique Provider Identification Number	Categorical
NCH_PRMRY_PYR _CLM_PD_AMT	NCH Primary Payer Claim Paid Amount	Numerical
AT_PHYSN_NPI	Attending Physician - NPI - Number	Categorical
OP_PHYSN_NPI	Operating Physician - NPI - Number	Categorical
OT_PHYSN_NPI	Other Physician - NPI - Number	Categorical
CLM_UTLZTN _DAY_CNT	Claim Utilization Day Count	Numerical
ADMTNG_IDC9 _DGNS_CD	Claim Admitting Diagnosis Code	Categorical
CLM_DRG_CD	Claim Diagnosis Group Code	Categorical
ICD9_PRCDR_CD_1	Claim Procedure Code 1	Categorical

Inpatient dataset of DE-SynPUF contains the 10 features for Beneficiary Diagnosis, that are excluded since dataset also contains $Claim_Admitting_Diagnosis_Code$ which indicates the beneficiary’s initial diagnosis at the time of admission. Mostly the claim is done through this one feature, rest of the diagnosis codes are mainly used for side diseases. Similarly, the procedure code has 6 features but only $Claim_Procedure_Code_1$ is used. Rest of the procedure code features contains the same code as diagnosis code. After the selection of the features, the values within every feature was labelled in such a way that help distinguish the code after the generation of the rules though association rule mining as shown in the Table 3.

### Findings and interpretations

Two experiments are conducted in this study. Initially, all baseline anomaly detection techniques are applied on the preprocessed data. This approach was time intensive as presented in Table 4. In second experiment, frequent rules are mined using association rule mining, specifically, through apriori algorithm and then unsupervised techniques are applied on the extracted rules. The time consumed using this approach is presented in Table 5. The time delay seen through the comparison of the two approaches shows our approach performs better even when using 100% of the dataset. The achieved difference in time is due to the fact that when using conventional approach, the experiment needs to be repeated each time a new transaction is added to the database, however the proposed approach works by extracting rules from transactions once thereby training our model to classify new instances of transactions as fraudulent or non fraudulent.

**Table 4:** Unsupervised Techniques Applied Independently on the Dataset

Dataset / Detector	50%	75%	100%
IF	1.84 Sec	2.67 Sec	3.4 Sec
CBLOF	0.79 Sec	0.80 Sec	2.23 Sec
OCSVM	224.29 Sec	507.96 Sec	897.24 Sec
ECOD	0.6 Sec	1.03 Sec	1.39 Sec
**Total Time**	227.53 Sec	512.45 Sec	904.24 Sec

**Table 5:** Unsupervised Techniques Applied on the Rules Extracted from Apriori Algorithm on 100% of the dataset

Algorithm	Time
Apriori Algorithm	867.06 Sec
Apriori $+$ IF	0.08 Sec
Apriori $+$ CBLOF	0.08 Sec
Apriori $+$ OCSVM	0.08 Sec
Apriori $+$ ECOD	0.08 Sec
**Total Time**	868.18 Sec

The Apriori association rule mining algorithm when applied on the preprocessed dataset, results in 72 rules that frequently appear together in the CMS 2008-2010 DE-SynPUF dataset, presented in Table 6. Association rule mining seeks high-confidence rules. Confidence measures the strength of the association between two item sets, while support measures their frequency in the database. To evaluate the rules generated through Apriori association rule mining, the coverage score against every rule is calculated.

**Table 6:** Results of Apriori Association Rule Mining

SNO	Antecedent	Consequent	Support	Confidence	Lift	Leverage	Coverage
1	DC-7802	A5000	0.0169	0.6712	1.7176	0.0071	0.8545
2	DC-78650	A5000	0.0232	0.5732	1.4668	0.0074	0.8545
3	PC-3995	A10000	0.0106	0.5498	1.6892	0.0043	0.9484
4	2 Day[s]	A5000	0.0811	0.5482	1.4028	0.0233	0.8545
5	1 Day[s]	A5000	0.0626	0.5416	1.3859	0.0174	0.9202
6	PC-8154	A15000	0.0128	0.5188	3.73	0.0094	0.8545
7	PC-8154	3 Day[s]	0.012	0.4848	2.884	0.0078	0.8545
8	DC-4280	A10000	0.0122	0.4461	1.3706	0.0033	0.9108
9	3 Day[s]	A5000	0.0747	0.4443	1.1368	0.009	0.8451
10	DC-486	A10000	0.0155	0.4388	1.3482	0.004	0.9108
11	PC-9904	A10000	0.0122	0.4374	1.3438	0.0031	0.8451
12	DC-78605	A10000	0.0177	0.4326	1.3292	0.0044	0.9108
13	PC-9904	A5000	0.0117	0.4213	1.078	0.0008	0.8545
14	4 Day[s]	A5000	0.0495	0.4051	1.0366	0.0017	0.8451
15	DC-78605	A5000	0.0156	0.3797	0.9716	-0.0005	0.8545
16	DC-4280	A5000	0.0102	0.3712	0.95	-0.0005	0.8638
17	6 Day[s]	A10000	0.0254	0.3708	1.139	0.0031	0.8357
18	5 Day[s]	A10000	0.0339	0.3702	1.1372	0.0041	0.8545
19	5 Day[s]	A5000	0.0336	0.3672	0.9396	-0.0022	0.8451
20	DC-486	A5000	0.0129	0.3644	0.9324	-0.0009	0.8545
21	4 Day[s]	A10000	0.0444	0.3633	1.1162	0.0046	0.8545
22	8 Day[s]	A10000	0.0144	0.3585	1.1014	0.0013	0.8451
23	7 Day[s]	A10000	0.0191	0.3559	1.0933	0.0016	0.8451
24	3 Day[s]	A10000	0.0557	0.3312	1.0176	0.001	0.8451
25	6 Day[s]	A5000	0.0215	0.3145	0.8048	-0.0052	0.9108
26	2 Day[s]	A10000	0.0446	0.3016	0.9267	-0.0035	0.8545
27	1 Day[s]	A10000	0.0328	0.2838	0.872	-0.0048	0.8545
28	7 Day[s]	A5000	0.0151	0.2818	0.7211	-0.0058	0.8451
29	8 Day[s]	A5000	0.0102	0.2546	0.6514	-0.0055	0.8451
30	DC-78650	A10000	0.0101	0.2492	0.7655	-0.0031	0.9108
31	A5000	2 Day[s]	0.0811	0.2075	1.4028	0.0233	0.8545
32	A5000	3 Day[s]	0.0747	0.1911	1.1368	0.009	0.8451
33	A15000	3 Day[s]	0.025	0.1797	1.0689	0.0016	0.8545
34	A10000	3 Day[s]	0.0557	0.1711	1.0176	0.001	0.8545
35	A5000	1 Day[s]	0.0626	0.1601	1.3859	0.0174	0.8451
36	6 Day[s]	A15000	0.0108	0.158	1.1361	0.0013	0.9108
37	3 Day[s]	A15000	0.025	0.1487	1.0689	0.0016	0.8545
38	5 Day[s]	A15000	0.0127	0.1389	0.9988	0	0.8545
39	A10000	2 Day[s]	0.0446	0.1371	0.9267	-0.0035	0.9484
40	A10000	4 Day[s]	0.0444	0.1365	1.1162	0.0046	0.8451
41	4 Days	A15000	0.0166	0.1354	0.9737	-0.0004	0.8357
42	A5000	4 Day[s]	0.0495	0.1268	1.0366	0.0017	0.8451
43	A15000	4 Day[s]	0.0166	0.1191	0.9737	-0.0004	0.8545
44	A10000	5 Day[s]	0.0339	0.1041	1.1372	0.0041	0.8451
45	1 Days	A15000	0.0118	0.1021	0.7342	-0.0043	0.8451
46	A10000	1 Day[s]	0.0328	0.1008	0.872	-0.0048	0.8638
47	A15000	2 Day[s]	0.0138	0.0991	0.6697	-0.0068	0.8357
48	2 Day[s]	A15000	0.0138	0.0932	0.6697	-0.0068	0.8545
49	A15000	PC-8154	0.0128	0.0919	3.73	0.0094	0.8545
50	A15000	5 Day[s]	0.0127	0.0914	0.9988	0	0.8545
51	A5000	5 Day[s]	0.0336	0.086	0.9396	-0.0022	0.8357
52	A15000	1 Day[s]	0.0118	0.0848	0.7342	-0.0043	0.8638
53	A10000	6 Day[s]	0.0254	0.0779	1.139	0.0031	0.9014
54	A15000	6 Day[s]	0.0108	0.0777	1.1361	0.0013	0.8451
55	3 Day[s]	PC-8154	0.012	0.0711	2.884	0.0078	0.8451
56	A5000	DC-78650	0.0232	0.0592	1.4668	0.0074	0.8545
57	A10000	7 Day[s]	0.0191	0.0586	1.0933	0.0016	0.8451
58	A5000	6 Day[s]	0.0215	0.0551	0.8048	-0.0052	0.9014
59	A10000	DC-78605	0.0177	0.0545	1.3292	0.0044	0.8451
60	A10000	DC-486	0.0155	0.0477	1.3482	0.004	0.9202
61	A10000	8 Day[s]	0.0144	0.0441	1.1014	0.0013	0.9108
62	A5000	DC-7802	0.0169	0.0432	1.7176	0.0071	0.8545
63	A5000	DC78605	0.0156	0.0399	0.9716	-0.0005	0.8638
64	A5000	7 Day[s]	0.0151	0.0386	0.7211	-0.0058	0.9108
65	A10000	DC-4280	0.0122	0.0375	1.3706	0.0033	0.9108
66	A10000	PC-9904	0.0122	0.0375	1.3438	0.0031	0.8545
67	A5000	DC-486	0.0129	0.033	0.9324	-0.0009	0.8545
68	A10000	PC-3995	0.0106	0.0325	1.6892	0.0043	0.8545
69	A10000	DC-78650	0.0101	0.0309	0.7655	-0.0031	0.8451
70	A5000	PC-9904	0.0117	0.03	1.078	0.0008	0.8545
71	A5000	8 Day[s]	0.0102	0.0261	0.6514	-0.0055	0.9108
72	A5000	DC-4280	0.0102	0.026	0.95	-0.0005	0.8545

The rules only give information about the itemsets appearing together in the transaction, it does not identify the nature of the rule; thats is, if it is normal or fraudulent. To identify the nature of the generated rules, Isolation Forest algorithm is used over the rules. The Isolation Forest works by creating random decision trees to isolate fraudulent points from normal points in the dataset. The algorithm initially identified 14 fraudulent rules in the DE-SynPUF dataset. However, due to the sensitive nature of healthcare and financial transactions, three additional unsupervised algorithms named CBLOF, ECOD, and OCSVM are applied to obtain more reliable and weighted results.

**Fig. 15:**
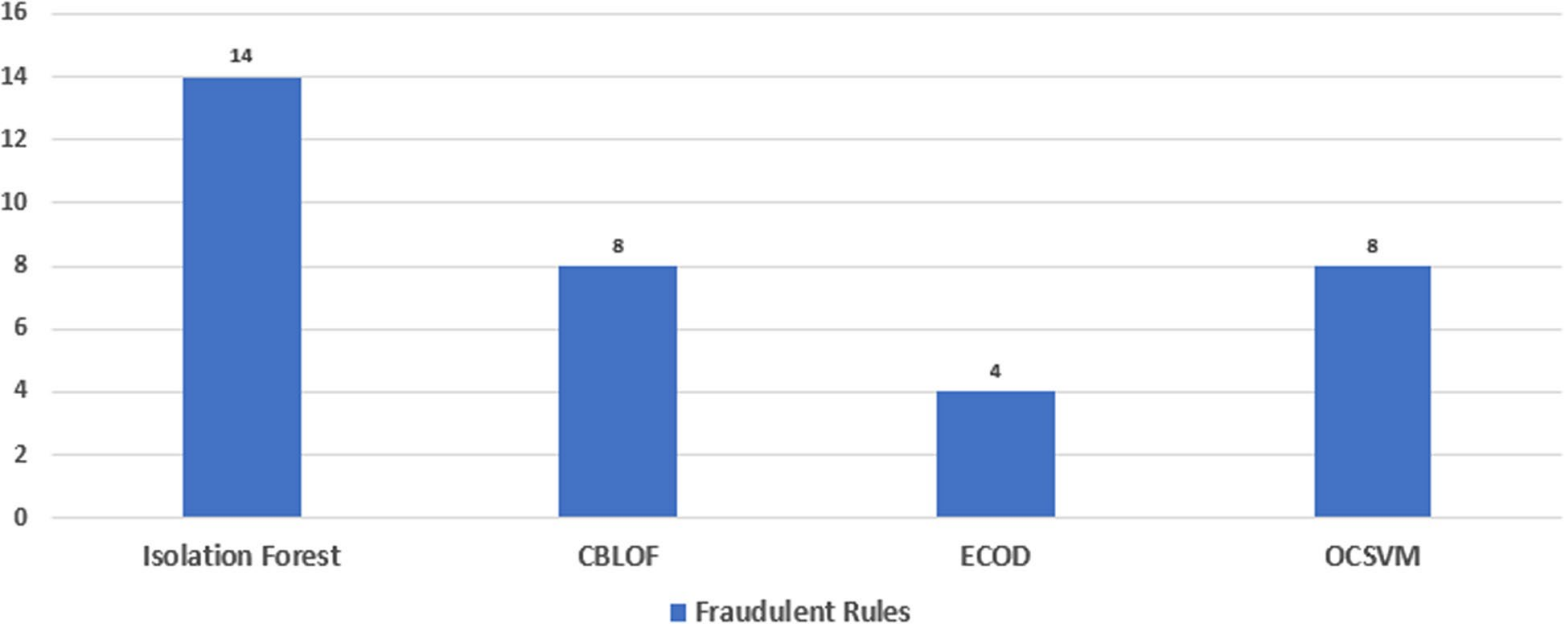
Fraudulent Classification Through Classifiers

As a result, the CBLOF, ECOD, and OCSVM algorithms identified 8, 4, and 8 fraudulent rules, respectively as shown in Fig. 15. In total, 52 out of 72 rules were marked as normal by all of the algorithms. However, in combination, 20 rules were marked as fraudulent by one or more algorithms.

**Table 7:** Rules Classification Through Applied Techniques

Rules classification	Rules
Normal Rules	52
Classified as Fraudulent By 1 Algorithm[s]	10
Classified as Fraudulent By 2 Algorithm[s]	6
Classified as Fraudulent By 3 Algorithm[s]	4
Classified as Fraudulent By 4 Algorithm[s]	0

**Table 8:** Identification of fraudulent rules through applied techniques

SNo	Rules	IF	CBLOF	OCSVM	ECOD
1	Diagnosis Code=7802 $\wedge$ Patient Pay Less Than 5000 USD	0	0	1	1
2	Procedure Code =3995 $\wedge$ Parient Pay More Than 5000 USD and Less Than 10000 USD	0	0	0	1
3	Procedure Code=8154 $\wedge$ Patient Pay More Than 10000 USD and Less Than 15000 USD	1	1	0	1
4	Procedure Code=8154 $\wedge$ Patient Stay is 3 Days	0	1	1	1
5	Patient Stay= 1 Day[s] $\wedge$ Parient Pay More Than 5000 USD and Less Than 10000 USD	1	0	0	0
6	Patient Stay =6 Day[s] $\wedge$ Patient Pay More Than 10000 USD and Less Than 15000 USD	1	0	0	0
7	Patient Stay=5 Day[s] $\wedge$ Patient Pay More Than 10000 USD and Less Than 15000 USD	1	0	0	0
8	Patient Stay=4 Day[s] $\wedge$ Patient Pay More Than 10000 USD and Less Than 15000 USD	1	1	0	0
9	Patient Pay More Than 10000 and Less Than 15000 USD $\wedge$ Patient Stay is 4 Days	1	1	0	0
10	Patient Stay=1 Day[s] $\wedge$ Patient Pay More Than 10000 USD and Less Than 15000 USD	1	0	0	0
11	Patient Pay More Than 5000 and Less Than 10000 USD $\wedge$ Patient Stay is 1 Days	1	0	0	0
12	Patient Pay More Than 10000 and Less Than 15000 USD $\wedge$ Patient Stay is 2 Days	1	1	0	0
13	Patient Stay=2 Day[s] $\wedge$ Patient Pay More Than 10000 USD and Less Than 15000 USD	1	1	0	0
14	Patient Pay More Than 10000 and Less Than 15000 USD $\wedge$ Procedure Code is 8154	1	1	0	1
15	Patient Pay More Than 10000 and Less Than 15000 USD $\wedge$ Patient Stay is 5 Days	1	0	0	0
16	Patient Pay More Than 10000 and Less Than 15000 USD $\wedge$ Patient Stay is 1 Days	1	0	0	0
17	Patient Pay More Than 10000 and Less Than 15000 USD $\wedge$ Patient Stay is 6 Days	1	0	0	0
18	Patient Stay=3 Day[s] $\wedge$ Procedure Code is 8154	0	1	1	1
19	Patient Less Than 5000 USD $\wedge$ Diagnosis Code is 7802	0	0	1	1
20	Patient Pay More Than 5000 and Less Than 10000 USD $\wedge$ Procedure Code is 3995	0	0	0	1

The results of our analysis are presented in Table 7, which shows the classification of rules according to the number of algorithms that classified them as fraudulent. The table indicates that 52 rules were classified as normal, while 10 were classified as fraudulent by one algorithm, 6 were classified as fraudulent by two algorithms, and 4 were classified as fraudulent by three algorithms . No rules were classified as fraudulent by all four algorithms.

These findings suggest that a combination of association rule mining and un-supervised classifiers help us achieve more reliable results in detecting healthcare insurance fraud. Detailed results against the transactions can be seen in Table 8. For example, The first rule states that if the Diagnosis Code is 7802 and the Patient Pay is less than $5000 USD, the claim is classified as fraudulent (1) by the One-OCSVM and ECOD, while it is classified as normal (0) by the Isolation Forest and CBLOF detectors. As for the second rule, it states that if the Procedure Code is 3995 and the Patient Pay more than $5,000 USD and less than$10,000 USD, the claim is classified as normal (0) by all detectors except ECOD. The following table shows the grammar along with the fraudulent status by the selected anomaly detection techniques.

The silhouette scores method is then applied to evaluate the effectiveness of four different anomaly detection techniques: Isolation Forest, CBLOF, ECOD, and OCSVM. The result of this evaluation is presented in Table 9. The silhouette scores for each technique are listed in the “Scores” column, while the “Classifier” column specifies the name of the anomaly detection technique used. As can be seen, the CBLOF technique has the highest silhouette score of 0.114, followed by Isolation Forest with a score of 0.103. The ECOD and OCSVM techniques have lower scores of 0.063 and 0.060, respectively.

**Table 9:** Silhouette Score for the Evaluation of Applied Techniques

No.	Classifier	Scores
1	Isolation Forest	0.103
2	CBLOF	0.114
3	ECOD	0.063
4	OCSVM	0.060

While comparing our study to the existing work on the same dataset, such as listed in Table 1, it is crucial to understand that the work presented in these studies is not transaction based and therefore cannot be compared at transactional level for fraud detection. In addition, process of feature engineering applied heavily relies on the available data sources, which limits the range of covariates linked to their single target variable, provider fraud, across different domains. For instance, while provider specialty is a common covariate in analyzing professional claims, it’s often overlooked in prescription claims analysis for different types of pharmacies. Our proposed approach adapts to the available features across various players and data sources within a domain. Our methodology is also capable of “graceful degradation,” meaning it continues to function when some data or variables are absent. Furthermore, the studies typically start with aggregated data before applying predictive algorithms, leaving the specifics of how certain claim components are aggregated rather vague. For instance, the method for aggregating variables such as the quantity dispensed and the compounding details in prescription claims is not well-defined. Lastly, the evaluation methods used are “error-based” instead of “cost-based”. Our presented approach extracts patterns or insights from disaggregated claims data, enhancing the current fraud literature and presenting a methodology evaluated on cost-based metric (coverage) making it suitable for practical investigative use.

There are, however, certain limitations in the presented work regarding the dataset diversity used for healthcare insurance fraud detection. While the dataset provided valuable insights into fraudulent patterns among patients, physicians, and services, it may not have fully captured the diversity of fraud scenarios prevalent in real-world settings. This limitation could potentially lead to biased or incomplete fraud detection model, as certain types of fraud or unique patterns may not have been adequately represented in the dataset. To address this limitation future work focuses on exploring techniques such as data augmentation to enrich the existing dataset. This could involve generating synthetic data points or incorporating external data sources to introduce more variability and complexity into the dataset.

The findings demonstrate the effectiveness of data mining techniques for healthcare insurance fraud detection that can have important implications for fraud prevention efforts in the healthcare industry. Further research can explore the temporal aspect of fraud patterns by conducting a thorough temporal analysis. This involves examining historical data to identify trends and changes in fraudulent behavior over time. By understanding how fraud patterns evolve and adapt, researchers can develop dynamic fraud detection models that can effectively detect emerging fraud schemes.

## Conclusion

The complexity and substantial monetary value of the healthcare industry makes it a desirable target for fraudulent activity. Due to the growing older population, healthcare insurance has been a consistent focus. The Centers for Medicare & Medicaid Services (CMS) and other organizations work ceaselessly to reduce fraudulent operations. The use of publicly accessible healthcare insurance data to identify and prevent potential fraudulent actions is a recent development, despite the issue’s longevity. Effective machine learning solutions can drastically minimize fraudulent occurrences and the resources necessary to investigate probable fraud cases.

In this study, a methodology based on combination of pattern recognition through association rule mining and unsupervised learning techniques is presented for detecting healthcare insurance fraud. Apriori association rule mining technique is used,that is not previously used on CMS 2008-2010 DE-SynPUF dataset. Rules obtained are further provided to the anomaly detection algorithms such as Isolation Forest, OCSVM, ECOD, and CBLOF. After combining all results, 20 rules are classified as fraudulent by one or more than one algorithms, and 52 are marked as normal.

The presented study shows promising results in detecting healthcare insurance fraud through identified methodology and provides a strong foundation for future research in the detection of healthcare insurance fraud using unsupervised learning techniques. The work is intended to continue towards improving the performance and developing a more comprehensive and effective framework for detecting fraudulent activities in healthcare insurance datasets.

## Data Availability

No datasets were generated or analysed during the current study.
